# Natural *GmACO1* allelic variations confer drought tolerance and influence nodule formation in soybean

**DOI:** 10.1007/s42994-024-00160-w

**Published:** 2024-04-18

**Authors:** Zhifang Zhang, Junkui Ma, Xia Yang, Shan Liang, Yucheng Liu, Yaqin Yuan, Qianjin Liang, Yanting Shen, Guoan Zhou, Min Zhang, Zhixi Tian, Shulin Liu

**Affiliations:** 1 Key Laboratory of Seed Innovation, Institute of Genetics and Developmental Biology, Chinese Academy of Sciences, Beijing, 100101 China; 2 The Industrial Crop Institute, Shanxi Agricultural University/Shanxi Academy of Agricultural Sciences, Taiyuan, 030031 China; 3 University of Chinese Academy of Sciences, Beijing, 100049 China

**Keywords:** Soybean, Drought tolerance, GWAS, Ethylene biosynthesis, Nodule formation

## Abstract

Soybean [*Glycine max* (L.) Merr.] is one of the most important, but a drought-sensitive, crops. Identifying the genes controlling drought tolerance is important in soybean breeding. Here, through a genome-wide association study, we identified one significant association locus, located on chromosome 8, which conferred drought tolerance variations in a natural soybean population. Allelic analysis and genetic validation demonstrated that *GmACO1*, encoding for a 1-aminocyclopropane-1-carboxylate oxidase, was the causal gene in this association locus, and positively regulated drought tolerance in soybean. Meanwhile, we determined that *GmACO1* expression was reduced after rhizobial infection, and that *GmACO1* negatively regulated soybean nodule formation. Overall, our findings provide insights into soybean cultivars for future breeding.

**Supplementary Information:**

The online version contains supplementary material available at 10.1007/s42994-024-00160-w.

Dear Editor**,**

Water is crucial for plant survival. Over the past 100 years, global water use has increased some six-fold, with an obvious consequence of a significant decrease in river water, on different continents, and increasing water deficit has exacerbated the frequency of drought disasters (Ault 2020). Soybean [*Glycine max* (L.) Merr.] was domesticated from wild soybean (*Glycine soja* Siebold & Zucc.) in China approx. 5000 years ago (Liu et al. 2020). However, soybean is one of the most drought sensitive crops, which can substantially reduce its yield and quality (Du et al. 2020a, 2020b). Therefore, breeding for high-yield, drought-tolerant soybean is crucial to meet the increasing demand for soybean production and cope with worsening water deficit.

Ethylene is a gaseous hormone involved in multiple aspects of plant metabolism, especially in response to abiotic stress (Wang et al. 2002; Xu and Zhang 2014. The biosynthesis of ethylene involves two steps: firstly, S-adenosylmethionine (SAM) is transformed into 1-aminocyclopropane-1-carboxylic acid (ACC, the precursor of ethylene), via ACC synthase (ACS); subsequently, ACC oxidase (ACO) oxidizes ACC into ethylene. ACSs and ACOs are the primary regulatory points in ethylene biosynthesis. Previous studies established that ACSs are the key rate-limiting enzymes of ethylene biosynthesis; however, emerging evidence indicates that ACOs have an important role in ethylene production under certain physiological conditions (Chen et al. 2016; Zheng et al. 2020).

We previously conducted a genome-wide association study (GWAS) in a natural soybean population and identified two significant drought tolerance association loci, located on chromosomes 8 (Chr. 8) and 16 (Chr. 16), and the causal gene *GmPrx16* on Chr. 16 was described in detail (Zhang et al. 2024). However, the candidate gene located on Chr. 8 remained unknown. In this study, we analyzed the genes at this locus on Chr. 8 and revealed that allelic variations in *GmACO1* contributed to drought tolerance differentiation in the soybean germplasms. Overexpression of *GmACO1* improved drought tolerance, whereas RNA interference (RNAi) transgenic lines exhibited the opposite phenotype.

The interval of the association locus on Chr. 8 covered 41 genes, and 10 protein-coding genes had polymorphisms whose haplotypes were significantly associated with SDTI variations in this population (Fig. 1A, Table S1). Only *Glyma.08G029200* (named *GmACO1* in this study) expression was induced by 15% PEG solution treatment (Fig. 1B, Fig. S1A). *GmACO1* encoded an ACC oxidase, and had two protein domains (https://phytozome-next.jgi.doe.gov). Moreover, *GmACO1* was highly expressed in roots, especially in the vascular bundles of roots (Fig. S1B, C). We observed that GmACO1 showed higher similarity with other reported ACOs in different species (Fig. S2), indicating that GmACO1 may participate in ethylene biosynthesis.

**Fig. 1:**
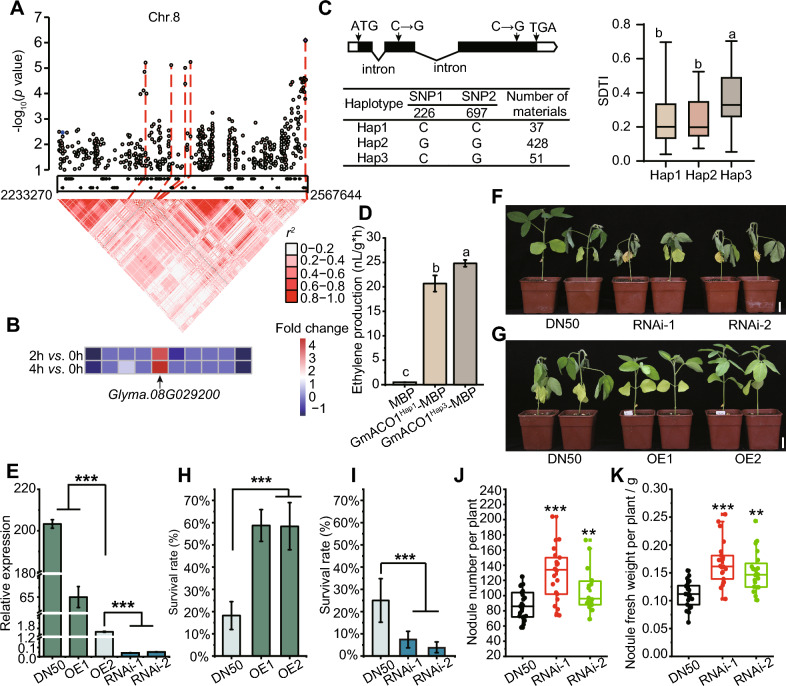
*GmACO1* improves drought tolerance in soybean. **A** Manhattan plot of the QTN on chromosome 8 (Chr. 8) (top) and linkage disequilibrium plot for SNPs within the genomic region around the QTN peak detected by GWAS. The color key (white to red) represents linkage disequilibrium values (*r*^*2*^). **B** Fold changes in the expression of the ten genes whose haplotypes were significantly associated with SDTI variation in this population after 15% PEG solution treatment for 2 h and 4 h compared with 0 h. **C** Gene structure and haplotype analysis of *GmACO1*. **D** GmACO1^Hap3^ exhibited increased oxidase activity. **E** *GmACO1* expression level in the transgenic lines and DN50. **F-G** Performance of RNAi transgenic lines (**F**) and overexpression transgenic lines (**G**) under drought conditions. Bar = 2 cm. OE1 and OE2 indicate *GmACO1* overexpression transgenic lines, and RNAi-1 and RNAi-2 indicate *GmACO1* RNAi transgenic lines. **H-I** Survival rates of *GmACO1* overexpression lines (**H**) and RNAi lines (**I**) after rehydration. **J** Nodule number per plant of DN50 and *GmACO1* RNAi transgenic lines at 28 dpi with *B. diazoefficiens* USDA110 (*n* > 18).** K** Nodule fresh weight per plant of DN50 and *GmACO1* RNAi transgenic lines at 28 dpi with *B. diazoefficiens* USDA110 (*n* > 18). The significance difference in C and D was calculated with one-way ANOVA with Tukey’s comparison, and the columns labeled with different letters are significantly different (*P* < 0.05); **, *P* < 0.01; ***, *P* < 0.001

Haplotype analysis revealed that two nonsynonymous SNPs existed in the predicted protein domain, and the Hap3 of *GmACO1* presented a significantly greater SDTI (Fig. 1C). We next investigated the oxidase activities of two GmACO1 haplotypes (Hap1 and Hap3), in vitro. These assays demonstrated that Hap3 showed higher oxidase enzyme activity than Hap1 (Fig. 1D).

To validate the function of *GmACO1* in conferring drought tolerance, we developed both overexpression (OE) of the CDS of Hap3 and RNAi on this gene in DN50 (an accession with Hap1). We obtained two independent lines for these OE and RNAi transgenic lines (Fig. 1E). Moreover, the amount of released ethylene differed in DN50 and *GmACO1* transgenic lines when the first ternate compound leaves expanded (Fig. S3A). Under well-watered conditions, no obvious phenotypic differences were observed among DN50 and these *GmACO1* transgenic lines (Fig. S3B, C). However, after withholding water for 12 days, the RNAi lines exhibited an obvious drought-induced wilting phenotype (Fig. 1F); after withholding water for 14 days, the OE lines exhibited a drought tolerant phenotype (Fig. 1G). After 14 days of drought treatment, we rewatered the plants and, subsequently, we observed that the survival rates for the OE lines were significantly higher than those of DN50 (Fig. 1H), while the survival rates for the RNAi lines were significantly lower compared with DN50 (Fig. 1I). These findings suggested that *GmACO1* was the causal gene in the QTN (quantitative trait nucleotides) located on Chr. 8, and it plays a positive role in conferring drought tolerance in soybean.

To further explore the putative pathways through which *GmACO1* may be involved in soybean drought tolerance, we performed RNA sequencing on *GmACO1* transgenic lines and DN50, under normal and drought conditions. We obtained 775 genes that were positively regulated with drought tolerance and 826 genes that were negatively correlated with drought tolerance (Fig. S4). Gene Ontology (GO) term enrichment analysis of these 775 DEGs and 826 genes suggested that these genes were enriched in multiple biological processes, including response to desiccation, plant hormone signal transduction and stomatal movement, negative regulation of gene expression, and DNA methylation (Fig. S5A, B). We randomly selected several genes, and determined their expression levels. The results confirmed the accuracy of the RNA sequencing data (Fig. S5C), suggesting that *GmACO1* affects multiple pathways involved in the response to drought tolerance.

Ethylene is a well-known gaseous plant hormone that is involved in multiple metabolic processes during plant development (Mao et al. 2016). Therefore, we investigated whether *GmACO1* has functions other than drought tolerance in soybean. Firstly, due to the high expression level of *GmACO1* in roots, we checked the root morphology of the transgenic lines and DN50; here no significant differences were observed between the *GmACO1* transgenic lines and DN50 (Fig. S6A).

Ethylene also acts as a negative regulator of rhizobial infection and inhibits the formation of nodules (Liu et al. 2018). Consequently, we checked the expression level of *GmACO1* after rhizobial infection and determined that it was reduced (Fig. S6B). Quantification of the total nodule number (per plant) and nodule fresh weight (per plant) at 28 days post inoculation (dpi), demonstrated that the *GmACO1* RNAi transgenic lines had more nodules than did the DN50 plants (Fig. 1J, K). However, the average nodule fresh weight was not significantly changed (Fig. S6C), suggesting that *GmACO1* negatively regulates nodule formation in soybean.

Taken together, we analyzed the genetic signal located on Chr. 8, and revealed that *GmACO1* was the causal gene for this QTN. Moreover, our transgenic experiments demonstrated that *GmACO1* could enhance drought tolerance in soybean. Thus, *GmACO1* provides a valuable genetic resource for developing drought tolerant soybean varieties, via molecular design breeding. Furthermore, our study also established that *GmACO1* can negatively regulate nodule formation, suggesting that this gene has multiple genetic effects. Consequently, *GmACO1* can be applied to soybean breeding for different purposes.

## Materials and methods

### Plant materials and phenotyping

The 585 soybean accessions used for the GWAS were planted at the experimental farm of the Industrial Crop Institute, Shanxi Agricultural University/Shanxi Academy of Agricultural Sciences, Fen-yang (37°15’N and 111°47’E), Shanxi Province, during the summer season in 2015. The materials were planted in irrigated and non-irrigated farm fields (natural drought stress treatment), and sump tanks were used in the non-irrigated farm fields to collect rainfall. A standard drought tolerance index was used to evaluate soybean drought tolerance, and cultivar Jindou No. 21 (JD21) was selected as the standard control. The formulas for calculation were as follows:

Drought tolerance coefficient (${\text{I}})=\frac{1}{n}\sum_{1}^{n}\frac{{x}_{1}}{{y}_{1}}$,

x, biomass, plant height and yield in the non-irrigated farm field;

y, biomass, plant height and yield in the irrigated farm field.

Drought tolerance index (B) = I × yield_drought_/yield_CK (JD21)_.

Standard drought tolerance index (SDTI) = B × 0.7371/I_CK (JD21)_.

### GWAS and permutation test for the drought tolerance index in soybean

Single-nucleotide polymorphisms (SNPs) with minor allele frequency (MAF) ≥ 0.05 and missing rate < 0.2 in the population from our 585 previously re-sequenced soybean accessions were used to perform GWAS for the SDTI (Fang et al. 2017). An association analysis was performed using a mixed linear model (MLM) implemented with the efficient mixed-model association expedited (EMMAX) software package (Kang et al. 2010). The threshold for GWAS was determined based on a previous report (Fang et al. 2017).

### Drought stress treatment

Two-week-old plants were subjected to the withholding water treatment. Each pot contained the same amount of soil (vermiculite: humus soil = 2:1), and the water supply was normal before drought stress treatment. When the first ternate compound leaves had expanded, the soil was fully watered, after which the water supply was stopped for approx. 14 days.

Three-week-old plants were used in the treatment with a 15% PEG solution to simulate drought. Only vermiculite was used in each pot. When the second ternate compound leaves had expanded, the plants were removed from the pots and thoroughly washed. Subsequently, the plants were subjected to the 15% PEG solution, after which the roots were sampled at different time points.

### RNA-seq and GO enrichment analysis

Two-week-old plants were subjected to the withholding water treatment. Total RNA was extracted from the ternate compound leaves of DN50 and transgenic plants under well-watered and drought conditions, with three biological replicates with two sets of comparison samples: DN50 versus transgenic plants under well-watered condition; DN50 versus OE1 under drought treatment for 12 d, and DN50 versus RNAi-1 under drought treatment for 10 d. Differentially expressed genes (DEGs) with a fold change ≥ 2 and a false discovery rate (FDR) < 0.05 were identified by edgeR. We calculated the fold changes in expression between the lines under normal and drought conditions; the results are shown as logFC_CK-DN50 *vs*. DR-DN50-OE_, logFC_CK-DN50 *vs*. DR-DN50-RNAi_, logFC_CK-OE1 *vs*. DR-OE1_, and logFC_CK-RNAi *vs*. DR-RNAi_. We divided the DEGs into two groups: the first group included the genes (logFC_CK-OE1 *vs*. DR-OE1_ > logFC_CK-DN50 *vs*. DR-DN50-OE_, and logFC_CK-DN50 *vs*. DR-DN50-RNAi >_ logFC_CK-RNAi *vs*. DR-RNAi_) that positively regulate drought tolerance affected by *GmACO1*; the other group included the genes (logFC_CK-OE1 *vs*. DR-OE1_ < logFC_CK-DN50 *vs*. DR-DN50-OE_ and logFC_CK-DN50 *vs*. DR-DN50-RNAi_ < logFC_CK-RNAi *vs*. DR-RNAi_) that negatively regulate drought resistance affected by *GmACO1*. The Gene Ontology (GO) enrichment test was performed by ClusterProfiler (Yu et al. 2012).

### Enzyme activity of the recombinant GmACO1 protein

The full-length cDNAs of *GmACO1*^Hap1^ and *GmACO1*^Hap3^ were cloned and inserted into the pMal-c2X vector. For GmACO1 protein expression, cultures were grown at 37 °C in LB medium to an OD_600_ of 0.8 before induction with 0.4 mM IPTG overnight at 18 °C. GmACO1-MBP proteins were purified with amylose resin. The activity of GmACO1^Hap1^ and GmACO1^Hap3^ was assessed, as previously described (Bulens et al. 2011). The primers used are listed in Table S2.

### Nodulation assays

Williams 82 plants were grown in pots containing vermiculite. One-week-old seedlings were inoculated with 30 mL *B. diazoefficiens* strain USDA110, suspended in distilled water (OD_600_ = 0.08), after which the roots were sampled at different time points after inoculation. *GmACO1* transgenic plants were planted in pots containing vermiculite. Then two-week-old plants were inoculated with 30 mL *B. diazoefficiens* strain USDA110 suspended in distilled water (OD_600_ = 0.08). Nodule phenotypes were evaluated at 28 dpi.

## Supplementary Information

Below is the link to the electronic supplementary material.

Supplementary file1 (DOCX 3196 KB)

Supplementary file2 (XLSX 15 KB)

## Data Availability

The raw sequence data reported in this paper have been deposited in the Genome Sequence Archive (Genomics, Proteomics & Bioinformatics 2021) in National Genomics Data Center (Nucleic Acids Res 2022), China National Center for Bioinformation/Beijing Institute of Genomics, Chinese Academy of Sciences (GSA: CRA013615 and CRA013621) that are publicly accessible at https://ngdc.cncb.ac.cn/gsa.
