# Supplementary material for: Natural GmACO1 allelic variations confer drought tolerance and influence nodule formation in soybean
Source: aBIOTECH. 2024 Apr 18;5(3):351–5. doi: 10.1007/s42994-024-00160-w (PMC11399508; doi:10.1007/s42994-024-00160-w)
Supplement: Supplementary file 1 — Supplementary file1 (DOCX 3196 KB) [file 42994_2024_160_MOESM1_ESM.docx]

**
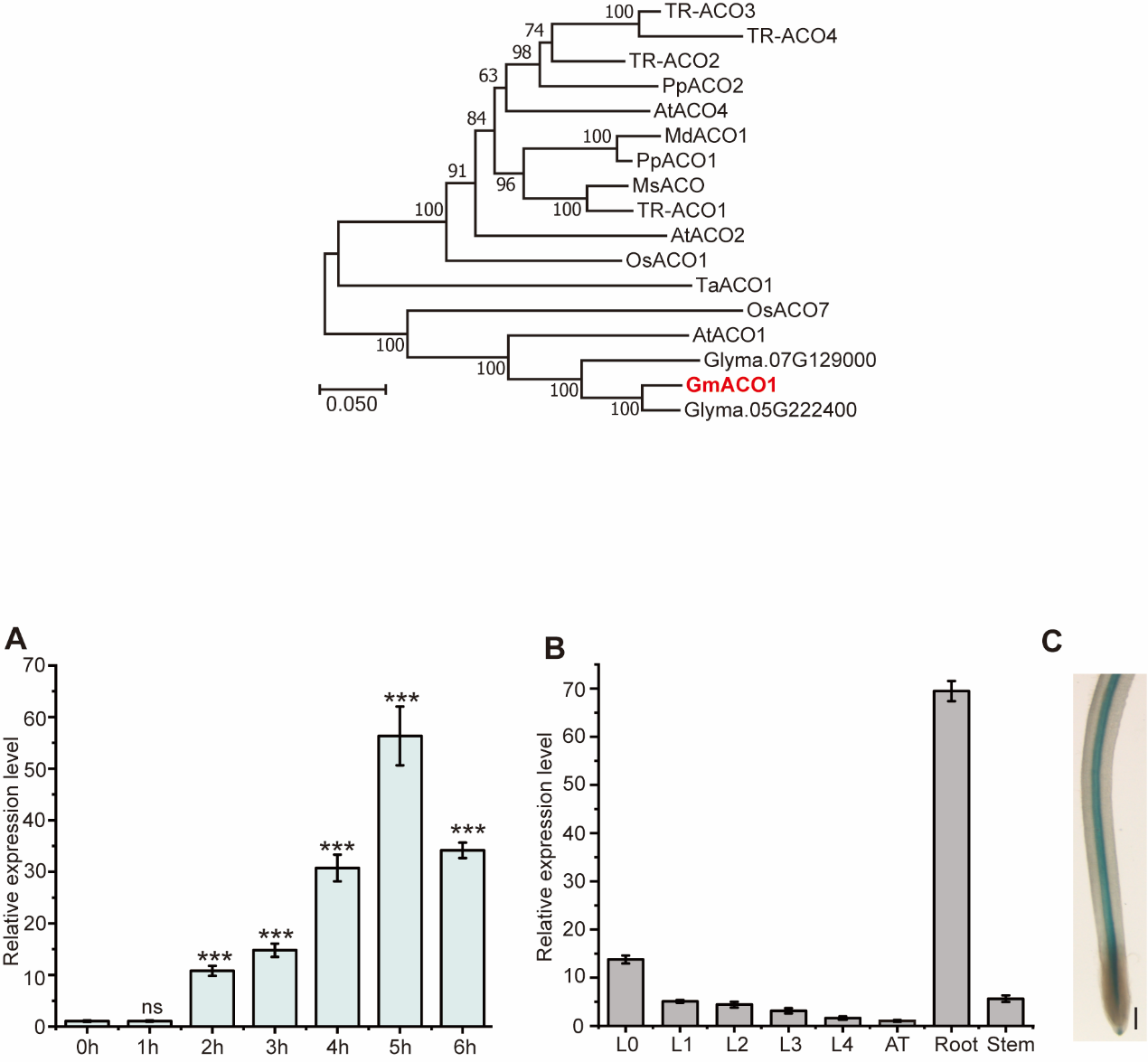
 Fig. S1** Expression pattern of *GmACO1*. **A** *GmACO1* was induced by 15% PEG solution treatment. ns: no significant difference, ***, *P* < 0.01. **B** Expression pattern of *GmACO1* in different plant tissues in DN50. L0 indicates the euphylla; L1-L4 denote the first, second, third and fourth ternate compound leaf, respectively; and AT indicates the apical tip. **C** *GmACO1* was primarily expressed in the vascular bundle in the root as determined by GUS staining. The 3 kb promoter of *GmACO1* was fused with the *GUS* reporter gene. Bar = 1 mm.


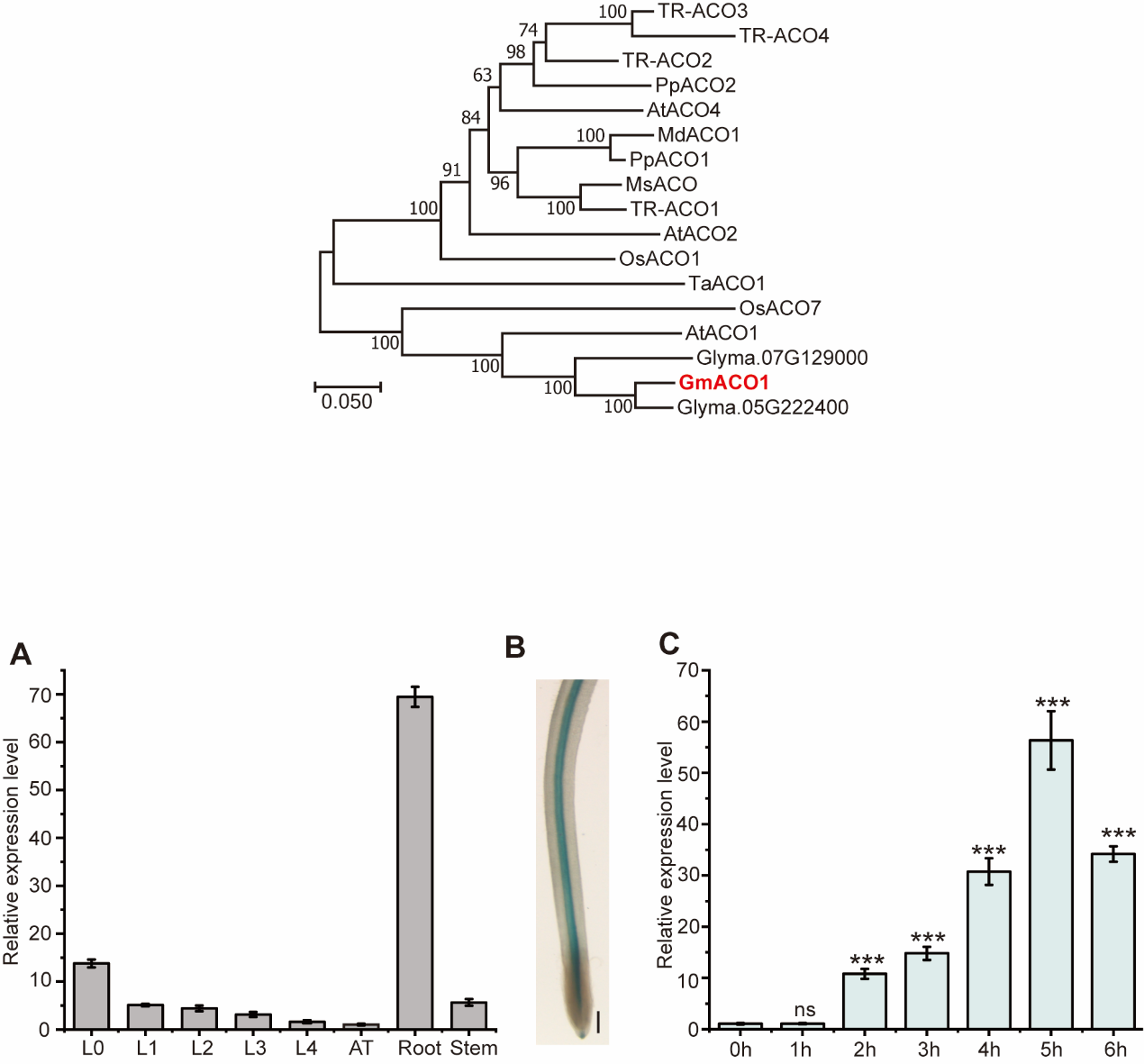


**Fig. S2** Phylogenetic analysis of GmACO1. Phylogenetic analysis of GmACO1 and its orthologs in different plants with reported oxidase enzyme activity. The phylogenetic analysis was performed using the neighbor joining method by MEGA7.


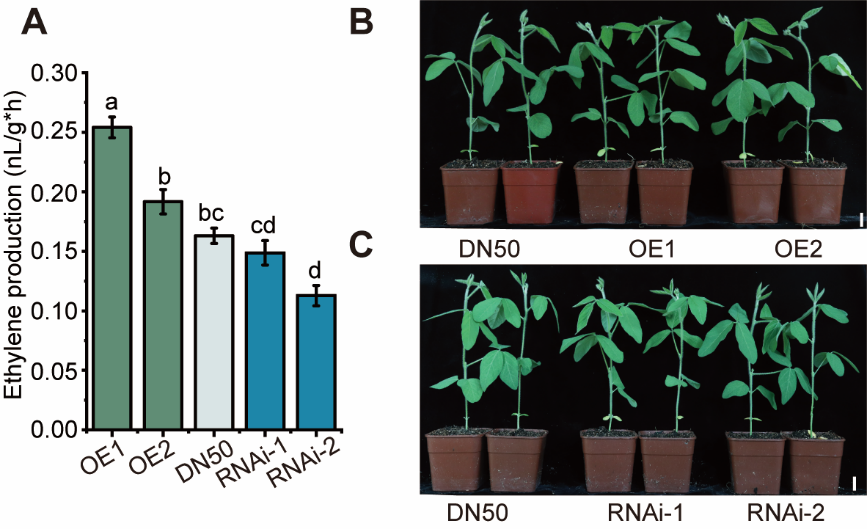


**Fig. S3** Functional analysis of *GmACO1* in soybean. **A** Ethylene production in *GmACO1* overexpression transgenic lines and RNAi transgenic lines and DN50. The significance of the difference was calculated with one-way ANOVA with Tukey’s comparison, and the columns labeled with different letters were significantly different (*P* < 0.05). **B-C** *GmACO1* overexpression transgenic lines (**B**) and RNAi transgenic lines (**C**) showed no significant difference under normal conditions. Bar = 2 cm.


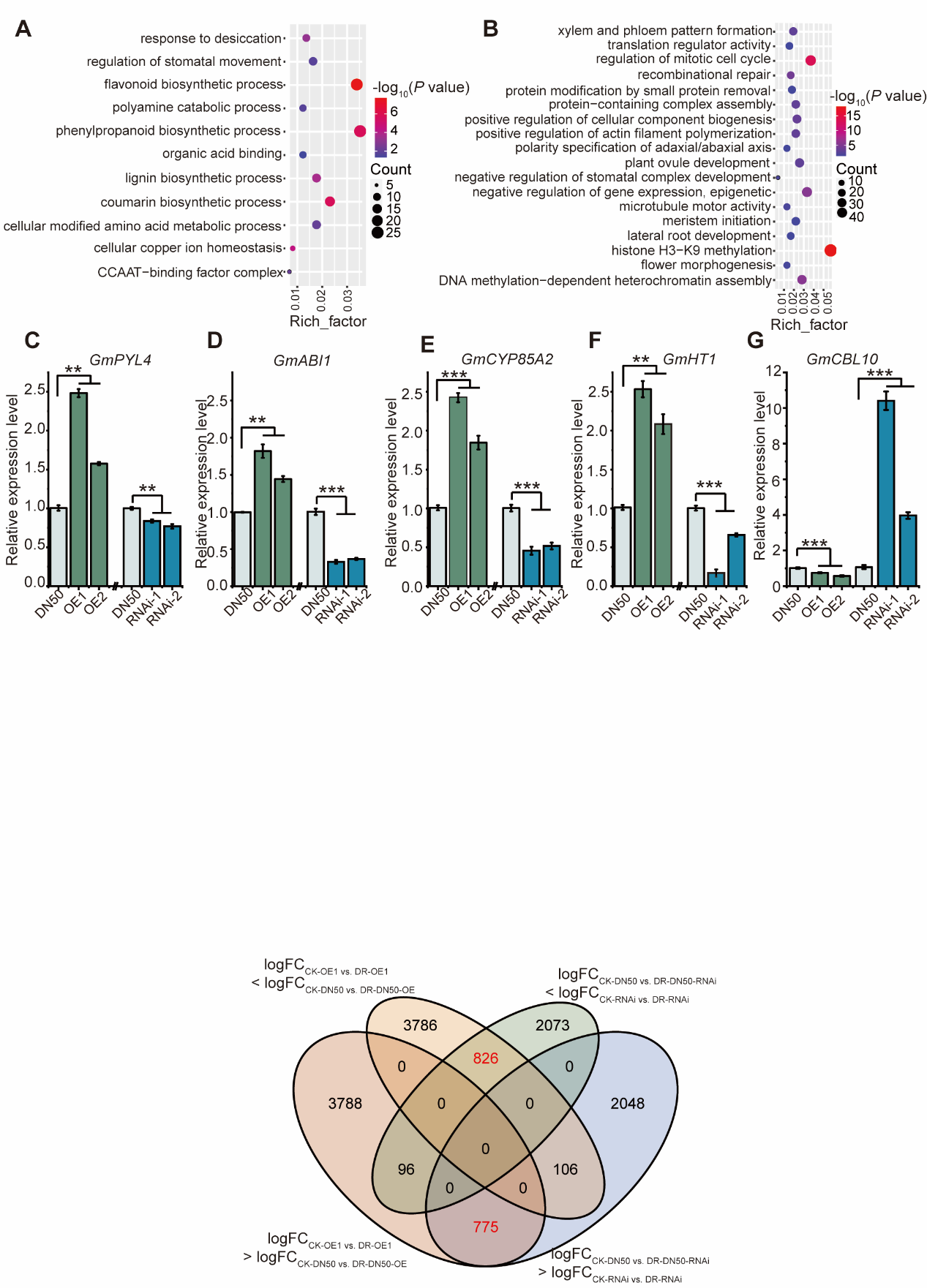


**Fig. S4** Venn diagram for the differentially expressed genes (DEGs) that positively or negatively regulate drought tolerance affect by *GmACO1*. The fold changes in expression between the lines under normal and drought conditions were marked as logFC_CK-DN50_ *_vs_*_. DR-DN50-OE_, logFC_CK-DN50_ *_vs_*_. DR-DN50-RNAi_, logFC_CK-OE1_ *_vs_*_. DR-OE1_, and logFC_CK-RNAi_ *_vs_*_. DR-RNAi_. 775 DEGs were positively regulated and 826 genes were negatively regulated by *GmACO1* under the imposed drought condition.


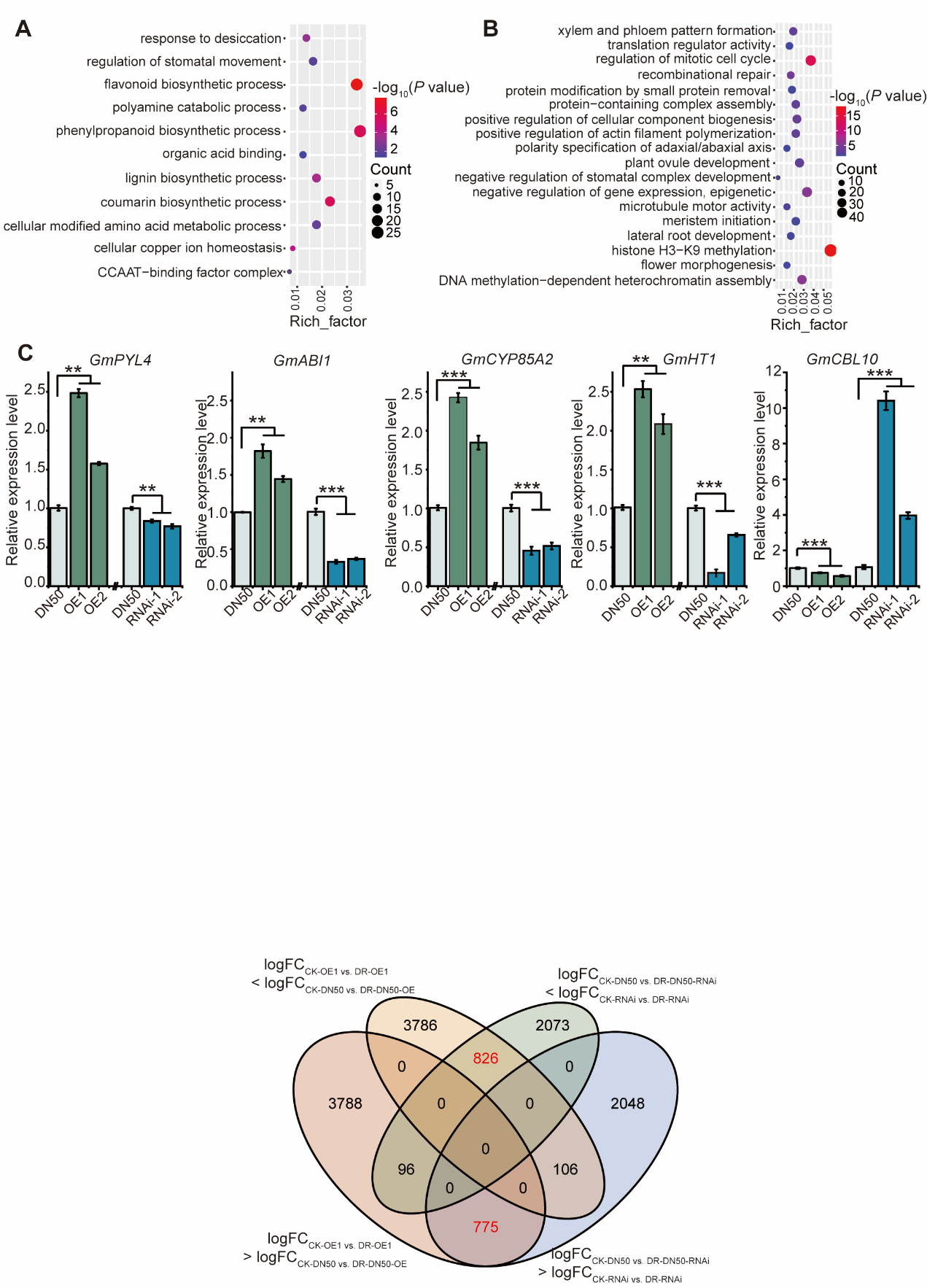


**Fig. S5** *GmACO1* influences multiple pathways under drought conditions. **A** GO enrichment of the genes that positively regulate drought tolerance affected by *GmACO1*. **B** GO enrichment of the genes that negatively regulate drought tolerance affected by *GmACO1*. **C** qRT-PCR results for genes involved in the drought response in soybean. *GmPYL4* and *GmABI1* are involved in ABA signaling, *GmCYP85A2* may affect brassinolide signaling, *GmHT1* affects the regulation of stomatal aperture, and *GmCBL10* is a member of the calcineurin B-like calcium sensor gene family. **, *P* < 0.01; ***, *P* < 0.001.


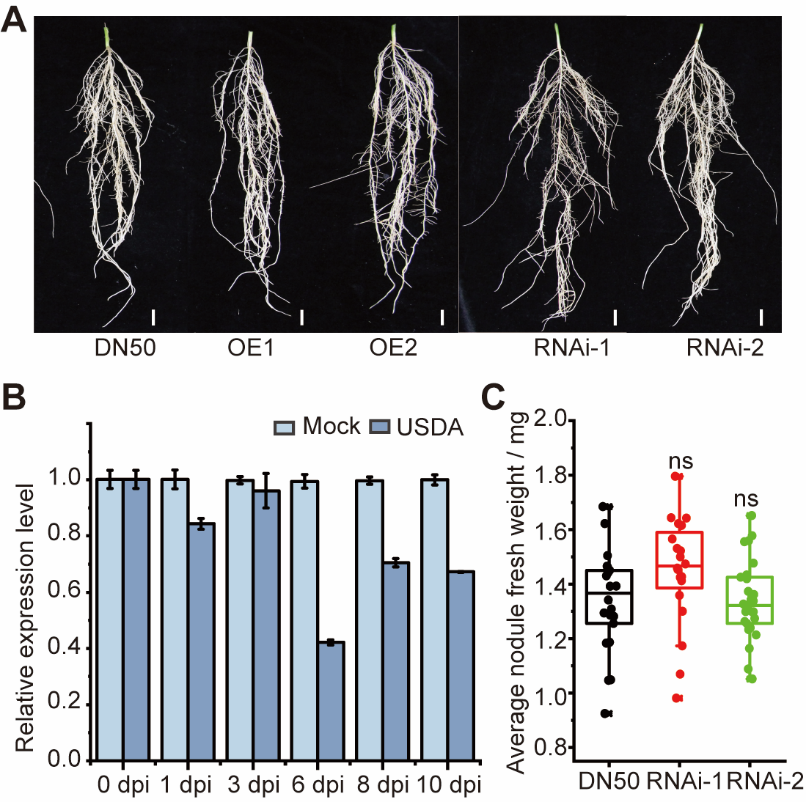


**Fig. S6** *GmACO1* inhibits nodule formation. **A** *GmACO1* had no effect on the morphology of soybean roots. Bar = 1 cm. **B** The expression level of *GmACO1* was inhibited at the early stages of nodule development. **C** Average fresh nodule weight per plant of DN50 and *GmACO1* RNAi transgenic lines at 28 dpi with *B. diazoefficiens* USDA110 (*n* > 18). ns indicates no significant difference; *, *P* < 0.05; **, *P* < 0.01.
